# Mathematical models with frills

**DOI:** 10.7554/eLife.48520

**Published:** 2019-06-25

**Authors:** Pierre A Haas

**Affiliations:** Department of Applied Mathematics and Theoretical Physics, Centre for Mathematical SciencesUniversity of CambridgeCambridgeUnited Kingdom

**Keywords:** *Chamydosaurus*, frilled dragon, elastic instability, patterning, morphogenesis, evolutionary developmental biology, Other

## Abstract

The spectacular frill around the neck of the lizard *Chlamydosaurus* has its origins in a mechanical instability that arises during development.

**Related research article** Montandon S, Fofonjka A, Milinkovitch M. 2019. Elastic instability during branchial ectoderm development causes folding of the *Chlamydosaurus* erectile frill. *eLife* **8**:e44455. doi: 10.7554/eLife.44455

The development of tissues and organs involves a combination of mechanical forces and various chemical and genetic cues (Mammoto et al., 2013; Nelson, 2016). A remarkable example of the role of mechanical forces during development is the formation of ridges and grooves (termed gyri and sulci) in the cerebral cortex to maximize its surface area-to-volume ratio. This process, which is called gyrification, does not require increased cell proliferation at the location of the gyri or other spatial patterning. Instead, it relies on a mechanical instability: the cerebral cortex (the outer layer of grey matter) grows uniformly, but it grows more than the underlying white matter to which it is attached, causing it to buckle and hence to form ridges and grooves (Figure 1; Tallinen et al., 2014; Nelson, 2016). An analogous mechanism of buckling due to differential growth has been proposed to explain the formation of the loops in the gut (Hannezo et al., 2011; Shyer et al., 2013).

Now, in eLife, Sophie Montandon, Anamarija Fofonjka and Michel Milinkovitch report that a similar instability plays a role in the development of the distinctive frill around the neck of *Chlamydosaurus kingii*, a lizard that is commonly called the ‘frilled dragon’ (Montandon et al., 2019). The frill is an outgrowth of cartilage and skin that, when erect for the purposes of defense or courtship, is rather akin to the ruffs that are found on the costumes of Elizabethan period dramas (Figure 1A). When at rest each of the two lobes of the frill settles into three folds (Figure 1B).

**Figure 1.**
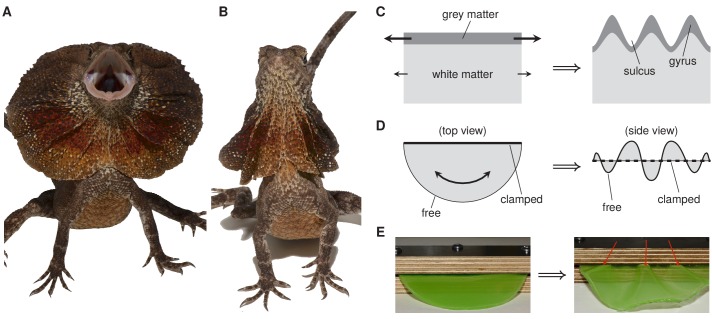
How frustrated growth can lead to the formation of frills. (**A**) *Chlamydosaurus kingii*, the frilled dragon, posturing with erect frill. (**B**) *Chlamydosaurus* with frill at rest. Each lobe of the frill has pleated into three folds. (**C**) During development of the cerebral cortex, grey matter grows more than white matter, leading to the formation of ridges and grooves called gyri and sulci. This is an example of frustrated growth at a surface. (**D**) The formation of the folds in the frill of *Chlamydosaurus* is an example of frustrated growth at a boundary: the frill is fixed at one edge by its attachment to the neck, so it buckles and forms folds as it grows. (**E**) Buckling due to boundary frustration illustrated by a physical analog experiment. Panels A, B and E are from Montandon et al., 2019.

Montandon et al., who are based at the University of Geneva and the SIB Swiss Institute of Bioinformatics, showed that the three folds appear on each lobe during embryonic development. However, staining for cell proliferation did not reveal any localized differences in cell division rates, so spatial patterning of cell proliferation cannot be responsible for the formation of the folds. Therefore the researchers hypothesized that the folds form due to the growth of the frill being frustrated by its attachment to the neck. A similar boundary frustration causes the folds of a draped curtain (Cerda et al., 2004).

To test their hypothesis, Montandon et al. performed an elegant experiment in which a semicircle of a gel (representing the frill) was first clamped between two blocks of wood (mimicking the attachment to the neck) and then immersed in a chemical. This caused the gel to swell, thus mimicking the growth of the frill (Figure 1E). The gel buckled to form three folds similar to those of the frill of *Chlamydosaurus.* The researchers then turned to numerical simulations of the frustrated growth of a thin elastic sheet, first in a simplified geometry, then in more realistic geometries, and were able to reproduce the transition from two folds to three folds that is seen during embryonic development in *Chlamydosaurus*.

The frills in *Chlamydosaurus* and the gyrification seen in the cerebral cortex both result from frustrated growth, though they differ in that the former involves frustration along an edge and the latter involves frustration along a surface. While the work of Montandon et al. highlights the importance of frustrated growth for morphogenesis, other researchers have shown that the gyrification observed in the cerebellum in mice, though superficially similar to that seen in the cerebral cortex, cannot be explained by the same model of frustrated growth (Engstrom et al., 2018).

The success of mechanisms of frustrated growth at explaining very different developmental processes (at least to a first approximation) suggests that continuum models (that is, models of continuous biological materials that average over different cells and other structures) have much to offer to the field of development. However, the occasional failure of continuum models emphasizes the importance of understanding the links between these models and the underlying biological processes.

An important challenge for these models is to move from qualitative comparisons between experimental systems and continuum models to more quantitative comparisons. Developing continuum models for this purpose again requires understanding how they link to the underlying biological processes. For example, continuum models often assume that Hooke's law is valid (that is, that the extension of the material is proportional to the force applied), but one would not expect this law to apply for large deformations of biological materials. Indeed, Hooke's law does not hold for brain tissue (Mihai et al., 2015). Theoretical work has shown how to extend Hooke's law to biological tissues by deriving the nonlinear continuum limit of simple cell-based models (Haas and Goldstein, 2019), and by using a framework of 'active gels' to describe biological materials that are out of equilibrium (Prost et al., 2015). However, the development of equations that can describe large deformations of general biological materials remains a long way away.
